# BMP2 and TGF-β Cooperate Differently during Synovial-Derived Stem-Cell Chondrogenesis in a Dexamethasone-Dependent Manner

**DOI:** 10.3390/cells8060636

**Published:** 2019-06-25

**Authors:** Nikolas J. Kovermann, Valentina Basoli, Elena Della Bella, Mauro Alini, Christoph Lischer, Hagen Schmal, Eva Johanna Kubosch, Martin J. Stoddart

**Affiliations:** 1 AO Research Institute, AO Foundation, 7270 Davos, Switzerland; niko2711@hotmail.com (N.J.K.); Valentina.basoli@aofoundation.org (V.B.); elena.dellabella@aofoundation.org (E.D.B.); mauro.alini@aofoundation.org (M.A.); 2 Equine Clinic, Free University of Berlin, 14163 Berlin, Germany; christoph.lischer@fu-berlin.de; 3 Department of Orthopaedics and Traumatology, Odense University Hospital, 5000 Odense, Denmark; hschmal@health.sdu.dk; 4 Department of Clinical Research, University of Southern Denmark, 5000 Odense, Denmark; 5 Department of Orthopedics and Trauma Surgery, Medical Center-Albert-Ludwigs-University of Freiburg, Faculty of Medicine, Albert-Ludwigs-University of Freiburg, 79106 Freiburg, Germany; johanna.kubosch@uniklinik-freiburg.de

**Keywords:** synovial derived stromal cell, BMP-2, TGF-β, dexamethasone, chondrogenesis

## Abstract

Recent studies highlighting mesenchymal stem cell (MSC) epigenetic memory suggest that a different differentiation medium may be required depending on the tissue of origin. As synovial-derived stem cells (SDSCs) attract interest we aimed to investigate the influence of TGF-β1, BMP-2 and dexamethasone on SDSC chondrogenesis in vitro. We demonstrate that dexamethasone-free medium led to enhanced chondrogenic differentiation at both the mRNA and matrix level. The greatest *COL2A1*/*COL10A1* ratio was detected in cells exposed to a combination medium containing 10 ng/mL BMP-2 and 1 ng/mL TGF-β1 in the absence of dexamethasone, and this was reflected in the total amount of glycosaminoglycans produced. In summary, dexamethasone-free medium containing BMP-2 and TGF-β1 may be the most suitable when using SDSCs for cartilage tissue regeneration.

## 1. Introduction

Articular cartilage (AC) is the most specialized connective tissue in diarthrodial joints, principally involved in facilitating physical movement and loading by limiting friction between bones due to its biological properties [1].

AC mainly consists of chondrocytes organized within a dense extracellular matrix (ECM) that is able to resist mechanical forces [2]. However, it is well known that AC has a limited capacity of self-regeneration upon injury due to its avascular microenvironment, and the limited number of chondrocytes that results in a low anabolic and biosynthetic ability [3]. Worldwide, traumatic injuries and degenerative cartilage defects such as osteoarthritis are major health problems that still do not have a definitive solution in terms of regeneration.

Current treatments possess several disadvantages associated with the limited availability of allograft or autologous tissue, and the low and restricted chondrocyte proliferative potential, which also seems to decrease with age [4]. Chondrocytes also dedifferentiate into a fibroblastic phenotype during in vitro culture [5]. To solve these problems an alternative treatment would be necessary.

Mesenchymal stromal cells (MSCs) are non-hematopoietic cells present in the niche of several adult tissues [6] that could represent an alternative cell source [7]. They have a degree of self-renewal capacity, in vitro chondrogenic potential [8] and relatively easy access [9]. Due to these properties, MSCs are considered a promising cell source for tissue engineering applications in the area of cartilage repair [10].

MSCs can be isolated from several tissues including bone marrow, fat [11], dental pulp [12], placenta [13], and also from synovial membrane [14]. Synovial-derived cells are increasingly being considered as good candidates for the repair of cartilage due to the simplicity of isolation and natural location within the articular joint [15] compared to cells from other sites. Earlier studies have shown that human synovial-derived stromal cells (hSDSCs) have a strong multilineage differentiation potential typical of MSCs, particularly toward the chondrogenic lineage [16]. Additionally, their hypertrophic and endochondral ossification potential is lower compared to MSCs derived from bone marrow (BMSCs) [14]. However, it is still necessary to carry out further studies before translating these cells into the clinic.

One of the biggest challenges in the field of regenerative medicine is to find a method that can guarantee a stable and reproducible differentiation [17]. For this reason, several research groups have focused their attention on the study of specific growth factors, chemical compounds and physical stimulation to induce stem-cell differentiation in a controlled manner. Of note, the protocols used for differentiation have largely remained unchanged with little additional optimization: adipogenic commitment requires the use of hydrocortisone, indomethacin, 3-isobutyl-1-methylxanthine, and dexamethasone [11]; osteogenic differentiation uses L-ascorbic acid 2-phosphate, beta-glycerol 2-phosphate, and dexamethasone [12], while chondrogenesis also requires L-ascorbic acid 2-phosphate [8] and dexamethasone, with additional TGF-β1. When considering the most commonly published differentiation protocols, the different cell sources are not often considered, and the standard protocols are applied regardless of cell source. This overlooks the fact that different cells, although commonly called MSCs can, and often do, respond differently to the compounds used for differentiation as they have pre-imprinted epigenetic and transcriptomic differences that make them differentially responsive to the environment [18,19]. This has led to an increased interest in tissue-specific MSC differentiation media.

Secondly, the use of dexamethasone, a glucocorticosteroid drug, is common in all differentiation protocols, independent of their commitment toward adipogenesis, osteogenesis or chondrogenesis. Dexamethasone is a synthetic glucocorticoid used in clinical practice for the treatment of inflammation and autoimmune conditions. It is used in the most common media for the induction of differentiation of mesenchymal stem cells toward the mesodermal lineage. Although glucocorticoids represent one of the most used medicaments for the treatment of many diseases, the limitation of this drug in the long term is due to its diverse (pleiotropic) effects, including potentially harmful side effect. After their diffusion through the cellular membrane due their lipophilic characteristic, glucocorticoids bind the respective glucocorticoid receptor, activating a downstream up-regulation of genes involved in anti-inflammatory responses. This occurs by directly modulating and binding DNA in the nucleus leading to gene transcription (a process known as transactivation). Alternatively, glucocorticoids can bind and repress the expression of proinflammatory proteins in the cytosol by preventing the translocation of NF-kB and its heteromeric binding proteins from the cytosol into the nucleus (transrepression).

However, there are few experimental studies comparing differentiation protocols specifically for synovium and fewer groups have investigated the role of dexamethasone during induction, modulation and differentiation in vitro. Recently, Shintai and Hunziker have shown that the effect of dexamethasone on chondrogenesis is dependent on cell source (tissue of origin and its microenvironment) and on the growth factor used [20].

Based on these observations, we investigated how hSDSCs respond to the conventional TGF-β1 chondrogenesis protocol [8] and how they respond to varying concentrations of TGF-β1 and BMP-2. These growth factors have already been shown to influence the expression of type II collagen and aggrecan in chondrocytes [21], the expression of chondrocyte-specific genes in bovine synovium-derived progenitor cells [22], and in human adipose-derived stem cells [23]. Additionally, the influence of the corticosteroid dexamethasone was investigated.

## 2. Materials and Methods

### 2.1. Isolation of Synovial-Derived Stem Cells

Chondrogenic differentiation was induced in human synovial derived stem cells isolated from synovial membrane (n = 4: male 42 y, male 41 y, male 19 y, male 54 y; obtained with full ethical consent approved by the Ethics Committee of the University of Freiburg as part of the “Tissue Bank for Research in the Field of Tissue Engineering” project (GTE-2002) and the biobank “Osteo” (AN-EK-FRBRG-135/14)). Samples were washed in 1:3 Betaisadona/phosphate-buffered saline (PBS) in Dulbecco’s modified Eagle medium containing 4.5 g/L glucose (DMEM-HG; Gibco, Thermo Fisher, Zürich, Switzerland) and then incubated in CollP-solution (1% collagenase) at 37 °C for 4 h.

After tissue digestion, the suspension cells were centrifuged at 500× *g* for 5 min, collected, and seeded in flasks for expansion with DMEM-HG.

hSDSCs were seeded at a density of 3000 cells/cm^2^ in DMEM-HG containing 10% MSC-qualified fetal bovine serum (FBS) (Pan Biotech, Aidenbach, Germany), 100  U/mL penicillin, 100 μg/mL streptomycin (Gibco, Thermo Fisher, Zürich, Switzerland), and 5 ng/mL recombinant human basic fibroblast growth factor (bFGF, Fitzgerald Industries International, Acton, MA, USA). Cells were cultured at 37 °C in a 5% CO_2_, 85% humidity atmosphere. Medium was changed every 2nd day until 70% confluence.

### 2.2. Induction of Chondrogenic Differentiation

Chondrogenic differentiation of hSDSCs between passage 3 and 4 was achieved using 3D pellet culture. 2 × 10^5^ hSDSCs per pellet were seeded in V-bottom 96-well plates (Corning, Corning, NY, USA) and centrifuged at 400× *g* for 5 min.

hSDSCs were committed towards the chondrogenic phenotype by switching to a chondrogenic medium, i.e., DMEM-HG, 1% non-essential amino acids (Gibco, Thermo Fisher, Zürich, Switzerland), 1% ITS+ (Corning), in the presence of 100 nM dexamethasone (Sigma-Aldrich, St. Louis, MO, USA), 5 µg/mL Ascorbic acid-2 phosphate (Sigma-Aldrich, St. Louis, MO, USA) and 10 ng/mL TGF-β1 (Fitzgerald). Other groups of cells were exposed to a lower concentration TGF-β1 (1 ng/mL) alone, or in the presence of BMP-2 at 1, 5, 10 ng/mL alone, or in double combination of 1 ng/mL TGF-β1 plus 1, 5, 10 ng/mL BMP-2; all the groups were cultured in the presence (+dexamethasone) or absence (-dexamethasone) of 100 nM dexamethasone. Every second day the media were replaced until day 21, when all the pellets were harvested for further analyses.

### 2.3. Real-Time Quantitative Polymerase Chain Reaction (PCR) Analysis

Total RNA was isolated from hSDSCs at day 0, before chondrogenic commitment, and after 21 days using TRI Reagent^®^ Solution (Molecular Research Centre Inc., Cincinnati, OH, USA) according to the manufacturer’s protocol. RNA quantity and quality were measured using the NanoDrop 1000 Spectrophotometer (Thermo Fisher, Zürich, Switzerland). For reverse transcription (RT) of 1 µg total RNA, TaqMan Reverse Transcription Kit (Applied Biosystems, Foster City, USA) was used. The RT reaction was carried out at 25 °C for 10 min, followed by 30 min at 42 °C and stopped by heating for 5 min at 85 °C. Relative gene expression (quantitative polymerase chain reaction (qPCR)) reactions were set up in 10 μL reaction mixtures containing TaqMan Universal Master Mix (Thermo Fisher, Zürich, Switzerland), the appropriate set of primers and probes, DEPC-H_2_O and cDNA template. The reaction program was set up as follows: 50 °C for 2 min, 95 °C for 10 min and 40 cycles of 95 °C for 15 s followed by an annealing/extension step at 60 °C for 1 min. All the qPCR runs were performed using StepOne Studio Real-Time PCR System (Thermo Fisher, Zürich, Switzerland). Technical triplicates were used for each target gene and for the different donors (biological replicates).

The relative expression of genes *COL2A1*, *COL10A1*, *ACAN*, *RUNX2*, *SOX9*, *SP7* (Osterix), *MMP13*, and *PPARG* during chondrogenic differentiation were determined using the 2^(-ΔΔCt)^ method, with ribosomal protein large, P0 (RPLP0) as reference gene and the day 0 sample (before chondrogenic commitment) as calibrator.

Primer and probe sequences are shown in supplemental Table S1 (supplementary material), while catalogue numbers of Assays-on-Demand (Applied Biosystems, Foster City, USA) are listed in the supplemental Table S2 (supplementary material).

### 2.4. Histological Staining Analysis

After 21 days in different culture media, samples were harvested and fixed in 70% methanol. One day before cutting, methanol solution was substituted with 5% sucrose and the samples were cryosectioned at constant thickness of 10 µm.

### 2.5. Safranin-O/Fast Green Staining

Safranin-O staining was performed on samples at day 21. The slides were washed in dH_2_O to remove the cryocompound, then stained with Weigert’s Haematoxylin solution (Sigma-Aldrich, St. Louis, MO, USA) for 10 min and washed in tap water for 10 min. The slides were then stained for 6 min with Fast Green (Fluka #51275) and for 15 min with Safranin-O (Sigma-Aldrich, St. Louis, MO, USA), followed by a wash with dH_2_O. After dehydration with increasing concentrations of ethanol, samples were transferred to xylene and coverslipped with Eukitt mounting medium (Sigma-Aldrich, St. Louis, MO, USA).

### 2.6. Immunofluorescence

After an initial wash in dH_2_O to remove the cryocompound, slides were transferred to methanol for 20 min. The non-specific binding sites were blocked with 10% FBS and PBS/Tween20 for 20 min. Primary antibody anti type II collagen (CIICI, see acknowledgement section) at a concentration of 5 μg/mL was added for 1 h at RT. Slides were washed with PBS, then the secondary antibody was added (Alexa Fluor 488 IgG 1:800) for 1 h at 37 °C. After washing with PBS, the nuclei were counterstained with 2-(4-Amidinophenyl)-1H-indole-6-carboxamidine (DAPI) 2.5 μg/mL and then coverslipped with Eukit mounting medium (Sigma-Aldrich, St. Louis, MO, USA).

### 2.7. Von Kossa Staining

Von Kossa staining for calcium and mineral deposition was performed on pellets at day 21. The slides were washed in dH_2_O to remove the cryocompound, then incubated for 30 min in a 5% silver nitrate solution (Fluka #85230) in direct sunlight. After rinsing in dH_2_O, slides were fixed with a 5% sodium thiosulfate (Fluka #72050) solution, rinsed again in dH_2_O and transferred to a nuclear fast red solution for counterstaining (Fluka #60700). After dehydration with increasing concentrations of ethanol, samples were transferred to xylene, and then coverslipped with Eukitt mounting medium (Sigma-Aldrich, St. Louis, MO, USA).

### 2.8. Macroscopic Evaluation

Morphological analysis was performed on uncut pellets to observe the possible shape and size differences among the groups. The pellets were collected on a glass side and covered with 100 μL of 70% methanol. A 2.5× objective on a Zeiss AxioPlan Microscope (Zeiss Microscopy GmbH, Jena, Germany) was used to take pictures of the pellets. Radius measurements were performed using Axiocam software (Zeiss Microscopy GmbH, Göttingen, Germany).

### 2.9. Glycosaminoglycan (GAG)/DNA Measurement

Pellet samples after 21 days were digested with 0.5 mg/mL proteinase-K (Sigma-Aldrich, St. Louis, MO, USA) at 56 °C overnight, followed by deactivation at 95 °C for 10 min. DNA content was measured with Hoechst 33,258 (Sigma-Aldrich, St. Louis, MO, USA) using a microplate reader (Victor3 Micro Plate Reader, Perkin Elmer, Waltham, MA, USA) with excitation at 360 nm and emission at 465 nm according to published methodology [24]. The amount of glycosaminoglycan (GAG) in the scaffolds and medium was determined by the dimethylmethylene blue dye method [25].

### 2.10. Statistical Analysis

Statistical analysis was performed using GraphPad Prism 7.03 software (GraphPad Software, San Diego CA, USA). Non-parametric two-way analysis of variance (ANOVA) in conjunction with Tukey’s multiple comparison test was applied. *p* < 0.05 was considered as statistically significant. A two-way ANOVA was used to evaluate distribution and homogeneity variance in the groups; the Tukey’s multiple comparison was used to evaluate the means of the different groups.

## 3. Results

### 3.1. Gene Expression Analysis

#### 3.1.1. TGF-β1 and BMP-2 Alone and in Combination Induces Chondrogenic Differentiation

To detect and clarify the role of two growth factors (TGF-β1 and BMP-2) during chondrogenic commitment of synovial-derived stem cells, both alone and in combination, the analysis of gene expression in pellet cultures was performed with qPCR.

**Figure 1:**
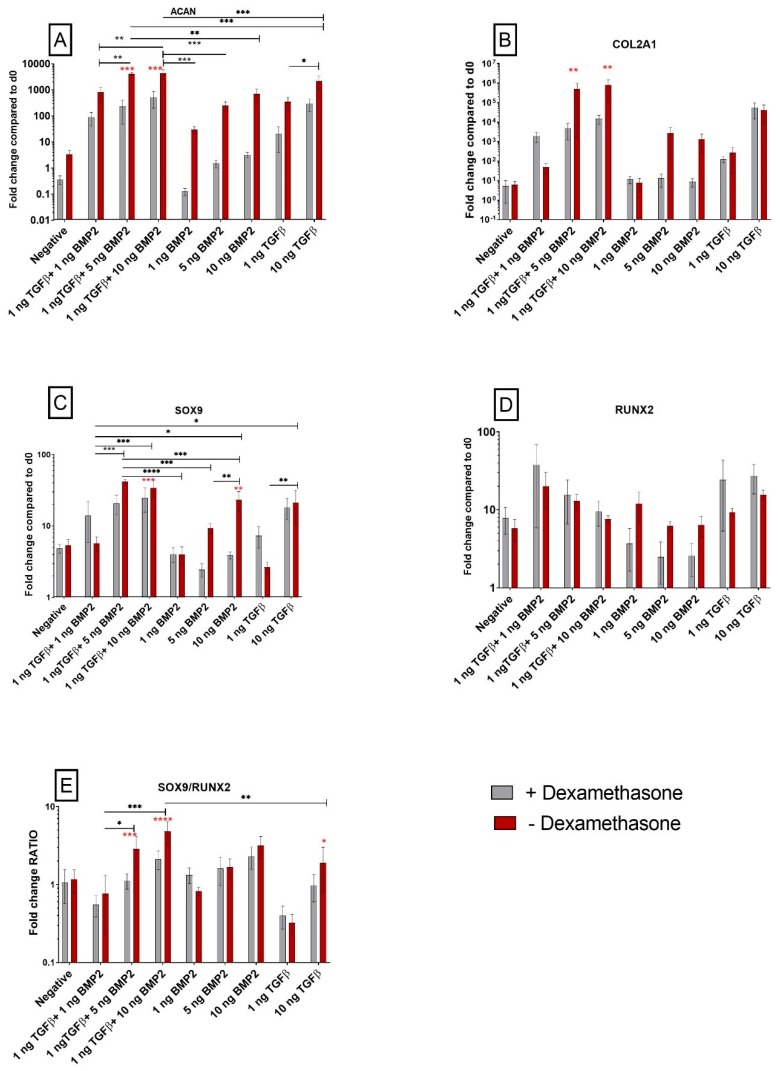
Effect of TGF-β1 and BMP-2 on the expression of genes orchestrating human synovial-derived stromal cell (hSDSC) chondrogenic commitment. Cells were induced to chondrogenic differentiation for 21 days in the presence (positive control) or absence (negative) of 10 ng/mL TGF-β1. Experimental groups contained low TGF-β1 concentrations (1 ng/mL TGF-β1), various concentrations of BMP-2 alone (1 ng/mL BMP-2, 5 ng/mL BMP-2, 10 ng/mL BMP-2), or BMP-2 in combination with 1 ng/mL TGF-β1 (1 ng/mL TGF-β1+1 ng/mL BMP-2, 1 ng/mL TGF-β1+5 ng/mL BMP-2 and 1 ng/mL TGF-β1+10 ng/mL BMP-2). The amounts of *ACAN* (**A**), *COL2A1* (**B**), *SOX9* (**C**), RUNX2 (**D**) mRNA were normalized to 60S acidic ribosomal protein P0 (*RPLP0*) and *SOX9*/*RUNX2* ratio (**E**) was calculated as the ratio between the fold change of each gene. The levels of gene expression of cells exposed to different media in the presence or absence of 100 nM dexamethasone were plotted as a fold change relative to the expression of the corresponding gene in undifferentiated cells (hSDSC day 0) defined as 1 (mean ± standard deviation (SD); n = 4). All data from 10 ng/mL TGF-β1 (positive control), low TGF-β1 concentration (1 ng/mL TGF-β1), and 1 ng/mL TGF-β1 combination with BMP-2 (1 ng/mL TGF-β1 + 1 ng/mL BMP-2, 1 ng/mL TGF-β1 + 5 ng/mL BMP-2 and 1 ng/mL TGF-β1 + 10 ng/mL BMP-2) were significantly different from negative control. Grey bars represent scatterplot of different measurements in hSDSC exposed to dexamethasone; red bars represent scatterplot of different measurements in hSDSC not exposed to dexamethasone. Data are expressed as mean ± SD, significant differences from dexamethasone treatment in the same group are marked by red asterisks; significant differences among group are marked by black asterisks (* *p* ≤ 0.05, ** *p* ≤ 0.01, *** *p* ≤ 0.001, **** *p* ≤ 0.0001).

The mRNA level of common markers associated with chondrogenic commitment during differentiation were significantly upregulated in all cells cultured with growth factors, independent of dexamethasone presence. Aggrecan (*ACAN*) expression was strongly modulated by TGF-β1 and BMP-2, with a *p* ≤ 0.001 compared to the negative control pellets that were not exposed to growth factors. The increase in *ACAN* expression was greater when the cells were not exposed to dexamethasone (Figure 1A). The combination of TGF-β1 (1 ng/mL) and BMP-2 (1 ng/mL, 5 ng/mL and 10 ng/mL) further increased *ACAN* mRNA expression.

Following the same pattern, collagen II (*COL2A1*) was upregulated in cells exposed to TGF-β1 alone, although the presence or absence of dexamethasone did not affect *COL2A1* expression when only TGF-β1 was used. Higher dose BMP-2, with or without TGF-β1, led to an increased *COL2A1* expression in the absence of dexamethasone when compared to dexamethasone-containing medium (Figure 1B).

#### 3.1.2. The *SOX9/RUNX2* Ratio as a Marker of Chondrogenic Potential of Human Synovial-Derived Stromal Cells (hSDSCs) In Vitro

To assess the chondrogenic potential of hSDSCs upon TGF-β1 and BMP-2 stimulation, the expression of two transcription factors involved in osteochondral fate was studied. Previous studies clearly showed that the *RUNX2*/*SOX9* ratio is a promising and early predictor marker for in vitro osteogenic potential of BMSCs [26,27]. *RUNX2* was largely unaffected by the different treatments (Figure 1D); however, while a low dose of TGF-β1 alone had little effect on *SOX9* expression, 10 ng/mL TGF-β1 led to its increase (Figure 1C). This suggests that during osteochondral fate a pivotal transcription factor modulated by TGF-β1 or BMP-2 is *SOX9*, which seems to be strongly affected using dexamethasone. In the absence of the glucocorticoid, the addition of BMP-2 led to a significantly increased *SOX9* expression. In the higher dose combination groups, the increases observed were less dependent on the presence of dexamethasone. *RUNX2* expression, however, was not controlled by TGF-β1 or BMP-2, suggesting a possible role of glucocorticoids action on the *SOX9*/*RUNX2* balance by way of changes in SOX9 expression (Figure 1E).

In the higher dose combination groups, the *SOX9*/*RUNX2* ratio was further enhanced when dexamethasone was not supplemented into the culture medium (Figure 1E).

#### 3.1.3. TGF-β1 Influences *COL10A1* Expression

**Figure 2:**
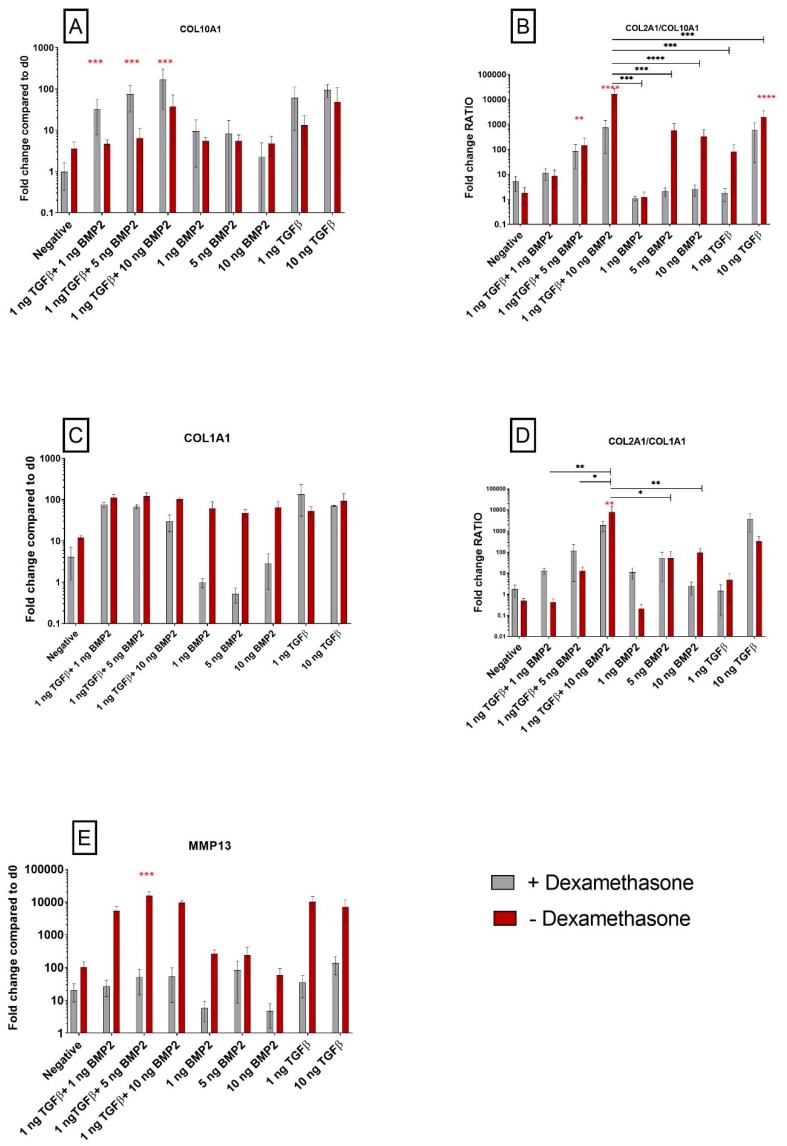
Dexamethasone modulates *COL10A1* expression, a hypertrophy marker, in hSDSCs exposed to TGF-β1 and BMP-2. Cells were cultured for 21 days in the presence of 10 ng/mL TGF-β1 (positive control) or in its absence (negative), low TGF-β1 concentration (1 ng/mL TGF-β1), different concentrations of BMP-2 alone (1 ng/mL BMP-2, 5 ng/mL BMP-2, 10 ng/mL BMP-2) or BMP-2 in combination with TGF-β1 (1 ng/mL TGF-β1 + 1 ng/mL BMP-2, 1 ng/mL TGF-β1 + 5 ng/mL BMP-2 and 1 ng/mL TGF-β1 + 10 ng/mL BMP-2). The amounts of *COL10A1* (**A**), *COL1A1* (**C**) and *MMP13* (**E**) mRNAs were normalized to *RPLP0*. The ratio between *COL2A1* and *COL10A1* (**B**) and *COL2A1* and *COL1A1* (**D**) was calculated as the ratio between the fold change of each gene. The levels of gene expression of cells exposed to different media in the presence or absence of 100 nM dexamethasone were plotted as a fold change relative to the expression of the corresponding gene in undifferentiated cells (hSDSC day 0) defined as 1 (mean ± SD; n = 4). Grey bars represent scatterplot of different measurement in hSDSC exposed to dexamethasone, red bars represent scatterplot of different measurement in hSDSC not exposed to dexamethasone. Data are expressed as mean ± SD, significant difference from dexamethasone treatment in the same group is marked by red asterisks; significant difference among group is marked by black asterisks (* *p* ≤ 0.05, ** *p* ≤ 0.01, *** *p* ≤ 0.001, **** *p* ≤ 0.0001).

Figure 1B shows *COL2A1* upregulation using TGF-β1 alone, but contrary to *ACAN* the presence or absence of dexamethasone did not affect its expression. With a similar trend, TGF-β1 alone increased *COL10A1* expression, however it was interesting to observe that *COL10A1* was further increased in dexamethasone-containing medium, while BMP-2 alone had little effect both in the presence or absence of dexamethasone (Figure 2A). The role of the glucocorticoid was most striking in the combination groups. When 1 ng/mL TGF-β1 was combined with either 1 or 5 ng/mL BMP-2, no increase in *COL10A1* was observed when dexamethasone was absent, but the levels of *COL10A1* increased in its presence. Even in the high-dose BMP-2 combination group, the presence of the glucocorticoid increased *COL10A1* expression over dexamethasone-free medium, suggesting an important role of dexamethasone in the determination of hypertrophy when TGF-β is present.

When comparing the *COL2A1*/*COL10A1* ratio, in order to establish the stability of chondrogenic differentiation, an increase was observed with 10 ng/mL TGF-β1 alone in either the presence or absence of the glucocorticoid (Figure 2B). With 1 ng/mL of TGF-β1 alone, the ratio was higher in the absence of dexamethasone. The absence of dexamethasone also leads to an improved *COL2A1*/*COL10A1* ratio, meaning that cells were expressing higher levels of *COL2A1* compared to *COL10A1* in the higher BMP-2 concentration groups. With the same trend, in the combination groups there was a dose-dependent increase in *COL2A1*/*COL10A1* ratio with increasing BMP-2 concentration, that was significantly enhanced by the absence of dexamethasone.

#### 3.1.4. Exposure to Corticosteroid Regulates *MMP13* Expression, while *COL1A1* Is only Influenced by BMP-2

Another important marker associated with chondrogenic differentiation is type I collagen, a major fibrillar component of undifferentiated mesenchymal progenitor cells and typical for fibrocartilage. Here we assessed the quality of chondrogenic differentiation and whether it was directed more into hyaline cartilage or to fibrocartilage using the *COL1A1* and *COL2A1*/*COL1A1* ratio as markers. In the groups containing TGF-β1, *COL1A1* expression increased over the negative control, with or without exposure to dexamethasone (Figure 2C). Interestingly, with the use of only BMP-2, *COL1A1* expression increased but only in the absence of dexamethasone. Overall, the *COL2A1*/*COL1A1* ratio was largely unaffected by dexamethasone and it reached the highest value in the group exposed to 1 ng/mL TGF-β1 + 10 ng/mL BMP-2 (Figure 2D).

Chondrogenic differentiation may also be monitored by assessing the expression of *MMP13* (Matrix Metallopeptidase 13), a gene that encodes for a protein that plays a role in the degradation of extracellular matrix proteins including fibrillar collagen, fibronectin and aggrecan. In this study dexamethasone inhibited the expression of *MMP13* under all conditions (Figure 2E). The differences in *MMP13* expression between dexamethasone-containing and dexamethasone-free media were most pronounced in groups containing TGF-β1. This suggests that during differentiation, in parallel with the expression of genes associated with a good quality of chondrogenesis, such as *COL2A1* and *ACAN* (Figure 1A–B), TGF-β1 induces the expression of *MMP13* and this can be attenuated by the use of dexamethasone.

#### 3.1.5. Dexamethasone Enhances Osteogenic Gene Expression when Combined with TGF-β1

**Figure 3:**
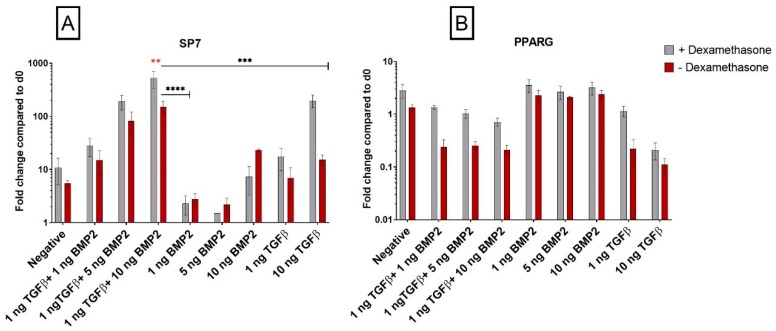
Dexamethasone modulates *SP7* (Osterix) and *PPARG* genes expression in hSDSCs exposed to TGF-β1 and BMP-2. Cells were cultured for 21 days in the presence of 10 ng/mL TGF-β1 (positive control) or in its absence (negative), low TGF-β1 concentration (1 ng/mL TGF-β1), various concentrations of BMP-2 alone (1 ng/mL BMP-2, 5 ng/mL BMP-2, 10 ng/mL BMP-2) or BMP-2 in combination with TGF-β1 (1 ng/mL TGF-β1 + 1 ng/mL BMP-2, 1 ng/mL TGF-β1 + 5 ng/mL BMP-2 and 1 ng/mL TGF-β1 + 10 ng/mL BMP-2). The amounts of *SP7* (**A**) and *PPARG* (**B**) mRNA were normalized to *RPLP0*. The levels of gene expression of cells exposed to different media in the presence or absence of 100 nM dexamethasone were plotted as a fold change relative to the expression of the corresponding gene in undifferentiated cells (hSDSC day 0) defined as 1 (mean ± SD; n = 4). Grey bars represent scatterplot of different measurement in hSDSC exposed to dexamethasone, red bars represent scatterplot of different measurement in hSDSC not exposed to dexamethasone. Data are expressed as mean ± SD; significant difference from dexamethasone treatment in the same group is marked by red asterisks; significant difference among groups is marked by black asterisks (* *p* ≤ 0.05, ** *p* ≤ 0.01, *** *p* ≤ 0.001, **** *p* ≤ 0.0001).

During mesodermal differentiation, cell fate could be easily affected by activation of unwanted pathways that decrease the yield and quality of differentiation. Since the likelihood of osteochondral differentiation at the expense of a stable chondrogenesis is high, we tested the expression of the osteogenic marker Osterix (*SP7*), a gene that encodes a member of the Sp subfamily of Sp/XKLF transcription factors. Sp family proteins are sequence-specific DNA-binding proteins essential for osteoblast differentiation [28]. The expression of *SP7* increased when 10 ng/mL TGF-β1 and dexamethasone were combined, while little change was seen in the absence of dexamethasone or at the lower TGF-β1 concentration (Figure 3A). BMP-2 alone had little influence of the expression of *SP7*. However, the combination of TGF-β1 and BMP-2 induced a dose-dependent increase in *SP7* expression that was lower in the absence of dexamethasone (Figure 3A). Therefore, dexamethasone increases the expression of *SP7* in the presence of TGF-β1, inducing an osteogenic or hypertrophic-like phenotype compared to cells in dexamethasone-free media.

#### 3.1.6. *PPARG* Is Regulated by TGF-β1

With the same principle, we further investigated if our differentiation protocol, and as well the use of dexamethasone, was influencing adipogenic differentiation of SDSCs. *PPARG* is a gene that encodes a member of the peroxisome proliferator-activated receptor (PPAR) subfamily of nuclear receptors and it is one of the principal regulators of adipocyte differentiation. The use of BMP-2 alone had no effect on *PPARG* expression (Figure 3B); on the other hand, TGF-β1 in the absence of dexamethasone strongly decreased *PPARG* expression, alone or in combination with BMP-2. This effect was more pronounced in the combination groups.

### 3.2. Biochemical Analyses

#### TGF-β1 Influences Pellet GAG Content

**Figure 4:**
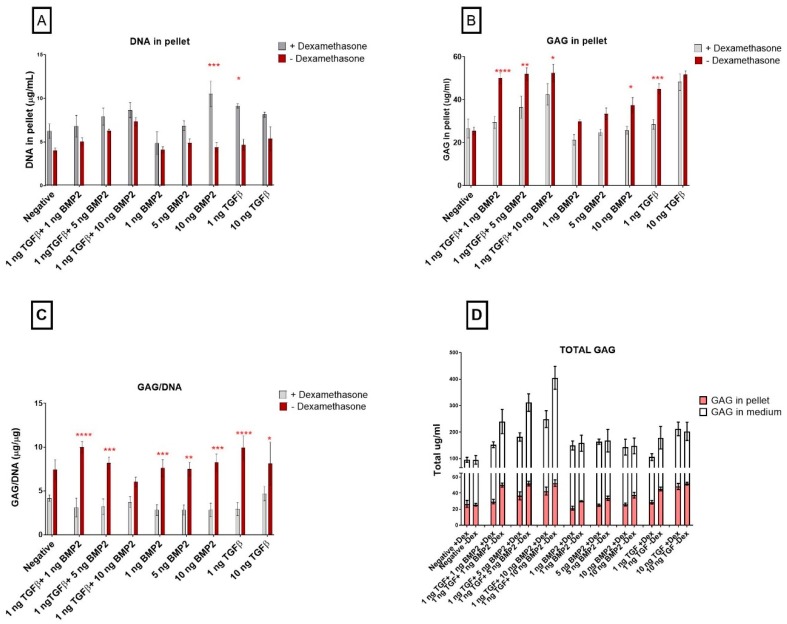
The effect of dexamethasone on glycosaminoglycan and DNA content in the pellets. Cells were cultured for 21 days in the presence of 10 ng/mL TGF-β1 (positive control) or in its absence (negative), low TGF-β1 concentration (1 ng/mL TGF-β1), various concentration of BMP-2 alone (1 ng/mL BMP-2, 5 ng/mL BMP-2, 10 ng/mL BMP-2) or BMP-2 in combination with TGF-β1 (1 ng/mL TGF-β1 + 1 ng/mL BMP-2, 1 ng/mL TGF-β1 + 5 ng/mL BMP-2 and 1 ng/mL TGF-β1 + 10 ng/mL BMP-2) (**A**). The amount of glycosaminoglycans in the pellets were normalized to the DNA content (**B**) (mean ± SD; n = 4). Total GAG is shown as GAG released in the medium (white bars) plus GAG retained inside the pellets (pink bars) (**C**). Grey bars represent different measurement in hSDSC exposed to dexamethasone, red bars different measurement in hSDSC in dexamethasone-free media. DNA content for pellets are shown in panel (**D**). Data are expressed as mean ± SD, significant difference from dexamethasone treatment in the same group is marked by red asterisks; significant difference among groups is marked by black asterisks (* *p* ≤ 0.05, ** *p* ≤ 0.01, *** *p* ≤ 0.001, **** *p* ≤ 0.0001).

Dexamethasone treatment generally led to an increased DNA content (Figure 4A) and this effect was more pronounced at the lowest TGF-β1 concentration (*p* ≤ 0.05) and as BMP-2 concentration increased. In the combination groups, there was a dose-dependent increase in DNA content (Figure 4A) and this was even more pronounced in the presence of dexamethasone. By day 21, combination group pellets cultured in dexamethasone-free media showed the highest GAG content (Figure 4B). However, GAG/DNA in the pellet was comparable between groups, with the absence of dexamethasone showing higher values (Figure 4C). The combination groups also demonstrated a higher GAG content in the absence of dexamethasone (Figure 4D).

### 3.3. Histological Analysis

#### 3.3.1. BMP-2 Modulates Pellet Size Differently in the Presence of Dexamethasone

**Figure 5:**
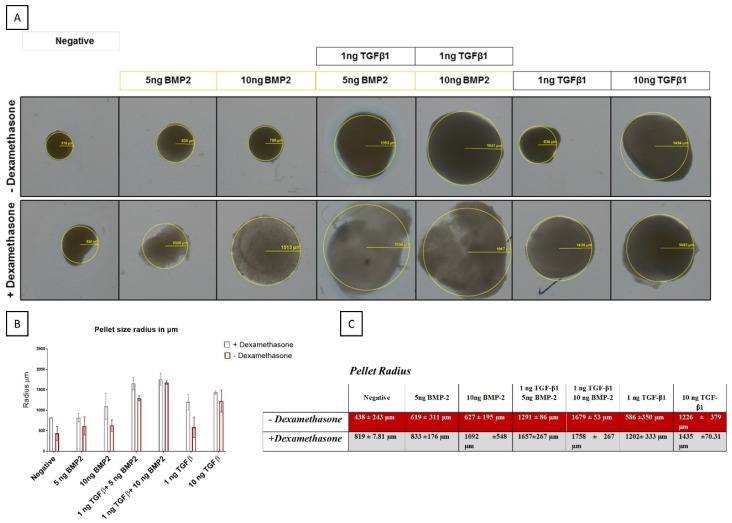
Macroscopic evaluation of pellet size from hSDSCs exposed to TGF-β1 and BMP-2 in the presence or absence of dexamethasone. hSDSCs in 3D culture were cultures for 21 days in the presence of 10 ng/mL TGF-β1 (positive control) or in its absence (negative), low TGF-β1 concentration (1 ng/mL TGF-β1), various concentrations of BMP-2 alone (5 ng/mL BMP-2, 10/mL ng BMP-2) or BMP-2 in combination with 1 ng TGF-β1 (1 ng/mL TGF-β1 + 5 ng/mL BMP-2 and 1 ng/mL TGF-β1 + 10 ng/mL BMP-2). The figures are representative of four separate experiments in four different donors (**A**). Radius sizes are expressed in µm (**B**–**C**). Radius pellet measured with Axio Plan Microscope objective 2.5×.

In general, the addition of dexamethasone in the differentiation media led to the formation of larger pellets, with only the 10 ng/mL TGF-β1 group being unaffected by glucocorticoid addition. Although smaller, pellets grown in the absence of dexamethasone appeared denser as determined by a decrease in light transmission (Figure 5).

#### 3.3.2. Safranin-O Staining Confirms Dexamethasone Influence during hSDSC Chondrogenic Differentiation

**Figure 6:**
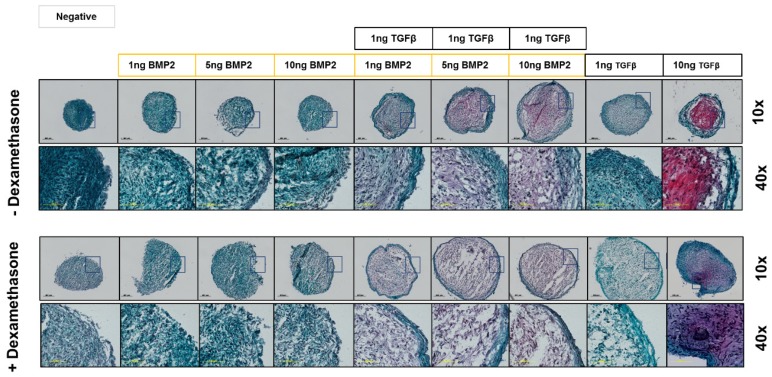
Safranin-O/Fast Green staining of hSDSC pellets exposed to TGF-β1 and BMP-2 in the presence or absence of dexamethasone. hSDSCs in 3D culture were cultured for 21 days in the presence of 10 ng/mL TGF-β1 (positive control) or in its absence (negative), low TGF-β1 concentration (1 ng/mL TGF-β1), various concentration of BMP-2 alone (1 ng/mL BMP-2, 5 ng/mL BMP-2, 10 ng/mL BMP-2) or BMP-2 in combination with 1 ng/mL TGF-β1 (1 ng/mL TGF-β1 + 1 ng/mL BMP-2, 1 ng/mL TGF-β1 + 5 ng/mL BMP-2 and 1 ng/mL TGF-β1 + 10 ng/mL BMP-2). The intensity of Safranin-O (Red) staining is directly proportional to the proteoglycan content inside the pellet, while green structures represent the counterstaining with Fast Green solution. The figures are representative of four separate experiments in four different donors. Black scale bar for 10× objective = 200 µm, yellow scale bars for 40× objective = 1000 µm.

To validate gene expression data at the matrix and protein level, pellets were stained on day 21 with Safranin-O/Fast Green. Pellets grown in conventional 10 ng/mL TGF-β1 media had increased GAG staining, with a more intense stain observed in the dexamethasone-free media. BMP-2 alone did not show any positive staining for GAG, either in the presence or absence of dexamethasone. In combination with TGF-β1, increasing BMP-2 concentration led to increased staining for GAG, an effect that was even more pronounced in the absence of dexamethasone (Figure 6).

#### 3.3.3. Anti-Collagen II Immunohistochemical Staining Confirms Dexamethasone Influence during hSDSC Chondrogenic Differentiation

**Figure 7:**
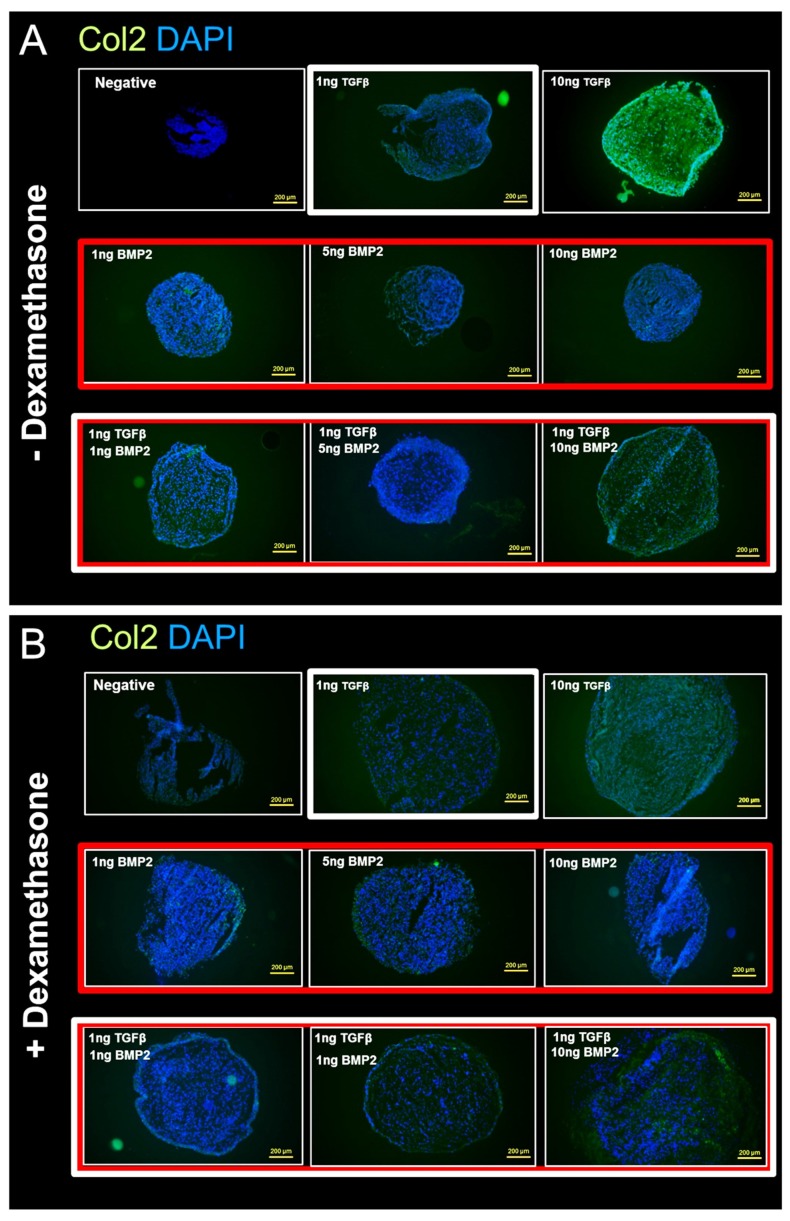
Immunofluorescence for type II collagen/DAPI on hSDSC pellets exposed to TGF-β1 and BMP-2 in the presence (**B**) or absence (**A**) of dexamethasone. hSDSCs in 3D culture were cultured for 21 days in the presence of 10 ng/mL TGF-β1 (positive control) or in its absence (negative), low TGF-β1 concentration (1 ng/mL TGF-β1), various concentration of BMP-2 alone (1 ng/mL BMP-2, 5 ng/mL BMP-2, 10 ng/mL BMP-2) or BMP-2 in combination with 1 ng/ml TGF-β1 (1 ng/mL TGF-β1 + 1 ng/mL BMP-2, 1 ng/mL TGF-β1 + 5 ng/mL BMP-2 and 1 ng/mL TGF-β1 + 10 ng/mL BMP-2). The intensity of type II collagen antigen reaction was comparable to the Safranin-O/Fast Green staining. Green color represents the positive reaction to type II collagen. Cells were stained blue with DAPI. The figures are representative of four separate experiments in four different donors. Yellow scale bar is 250 μm.

To detect and quantify the content of the type II collagen matrix deposition in hSDSC pellets, immunofluorescence staining for type II collagen was performed. The use of 10 ng/mL TGF-β1 without dexamethasone showed the strongest positivity for type II collagen (Figure 7A). Interestingly, compared to BMP-2 alone or to 1 ng/mL TGF-β1, the combination groups also showed a positive reaction, albeit not as strong as 10 ng/mL TGF-β1. Also, as expected, the pellets that were exposed to dexamethasone showed a weaker positive reaction with dexamethasone-free media, the same tendency that could be observed in the Safranin-O/Fast Green staining (Figure 7B).

#### 3.3.4. Von Kossa Staining Analysis for Calcium and Mineral Deposition

To detect and quantify the mineral and calcification deposition of the hSDSC pellets, von Kossa staining was performed. No mineralization or calcification could be detected in any group, indicating that the pellets were not undergoing calcification (data not shown).

## 4. Discussion

MSCs offer hope for tissue regeneration in difficult-to-replace tissues, such as cartilage. Recent studies have highlighted the role of epigenetic memory on MSC differentiation [29] and this suggests that cells from different sources may require differentiation media optimized for each cell source. The classical chondrogenic differentiation medium was optimized for bone marrow derived MSCs [8]. However, it has already been demonstrated that chondrogenesis of adipose-derived MSCs can be improved with the addition of BMP-6 [30]. Previous studies have also shown that different cells respond differentially to stimulation with the same growth factor [21]. Based on these observations, we evaluated how synovium-derived cells respond to TGF-β1, and BMP-2, and their combination, both in the presence or absence of dexamethasone.

Dexamethasone is a common factor present in all differentiation media, independent of induction toward chondrogenesis, adipogenesis or osteogenesis. This suggests that the use of dexamethasone is not a crucial committer towards a specific differentiation lineage, but a modulator during stem cell fate.

During chondrogenic differentiation, *ACAN*, *COL2A1* and *SOX9* were upregulated in the presence of the conventional concentration of TGF-β1. The combination of TGF-β1 and BMP-2 also led to chondrogenic differentiation. However, removal of 100 nM/mL dexamethasone improves chondrogenesis in the positive control (10 ng/mL TGF-β1) and in the TGF-β1/BMP-2 combination groups. Those data were confirmed via histological evaluation, which showed a higher positivity for GAG and type II collagen staining in cells not exposed to dexamethasone. These results are in line with our former findings where we could show a chondrogenic differentiation of SDSCs in a trans-well coculture of SDSC and chondrocytes induced by paracrine factors, such as TGF-β levels in the supernatants, without dexamethasone. The trans-well coculture of human synovial mesenchymal stem cells with chondrocytes leads to self-organization, chondrogenic differentiation, and secretion of TGF-β [31]. Previous studies have also shown the detrimental effect of 100 nM/mL dexamethasone on SDSC chondrogenic differentiation [32]. The same paper showed that 10 nM/mL dexamethasone also inhibited chondrogenesis when BMP-2 alone was used, compared to TGF-β alone [32]. The discrepancy between absolute levels of *ACAN* expression and the resulting GAG synthesis has been highlighted in previous studies yet the reason for the discrepancy is so far unknown [33,34].

Additionally, dexamethasone led to an increased expression of the hypertrophic marker *COL10A1*. Absence of the glucocorticoid led to a greater increase in *COL2A1* expression, therefore leading to a higher *COL2A1*/*COL10A1* ratio, suggesting a less hypertrophic phenotype. As hypertrophy (high collagen 10 and low collagen 2) is associated with a temporary cartilage template that will remodel into bone, a higher *COL2A1*/*COL10A1* ratio is considered beneficial for resting cartilage.

*MMP13* is a member of the matrix metalloproteinase family, that plays a role in the degradation of extracellular matrix proteins including collagen [35] and indicates a worse clinical outcome [36]. Dexamethasone clearly downregulated *MMP13* expression, most notably when TGF-β1 was present. *MMP13* is a hypertrophic marker [37] and is believed to be involved in the transition phase of cells toward an osteochondral fate [38] and has also been linked to osteoarthritis [39].

It is interesting to note that the GAG production in cells that were not treated with dexamethasone was significantly higher than in those treated with dexamethasone. Both GAG retention within the pellet and GAG release into the medium was higher when dexamethasone was absent, however in the combination group of BMP-2 and TGF-β1 there was less retention of GAG.

The influence of dexamethasone, BMP-2, TGF-β1, and their combination on other differentiation pathways was also investigated [40]. Osterix (encoded by *SP7* gene) is a transcription factor implicated in osteogenic differentiation, together with *RUNX2*, and it is upregulated by TGF-β1 also in BMSCs [41,42]. These factors are also implicated in hypertrophic differentiation during chondrogenesis, so further work would need to be performed to establish which pathway is being preferred. Dexamethasone had a limited effect on *SP7* expression, which was more regulated by growth factor combinations [43]. The influence of TGF-β1 and BMP-2 on adipogenic commitment was investigated using *PPARG* as a marker [44]. *PPARG* was largely unaffected by the conditions applied. However, there was a tendency towards a lower expression when dexamethasone was absent and TGF-β1 was present.

## 5. Conclusions

In conclusion, chondrogenic differentiation in the absence of dexamethasone leads to a more stable chondrogenic phenotype when hSDSCs are used as cell source. Therefore, based on the observed results, we suggest the use of a low TGF-β1 concentration in combination with BMP-2 to induce chondrogenic differentiation without the use of dexamethasone. As it has been demonstrated that kinematic load increases the production and activation of endogenous TGF-β1 [45,46], future clinical strategies might employ the implantation of SDSCs with BMP-2 (which is clinically approved) with the TGF-β1 component being generated endogenously by way of an optimised rehabilitation protocol. This could pave the way for the study of different methods for the differentiation of synovial cells, a relevant cell source that could be used in the field of regenerative medicine for patients with severe cartilaginous problems.

## Supplementary Materials

Supplementary tables are available online at https://www.mdpi.com/2073-4409/8/6/636/s1.
