# Supplementary material for: BMP2 and TGF-β Cooperate Differently during Synovial-Derived Stem-Cell Chondrogenesis in a Dexamethasone-Dependent Manner
Source: Cells. 2019 Jun 25;8(6):636. doi: 10.3390/cells8060636 (PMC6628125; doi:10.3390/cells8060636)
Supplement: Supplementary file 1 [file cells-08-00636-s001.pdf]

SUPPLEMENTARY FILE

Supplemental Table S1. Sequences of primers and probe used for qPCR. Probe modifications: FAM at 3' and NFQ-MGB at 5' end.

Gene	Forward primer	Reverse primer	Probe
RPLP0	TGGGCAAGAACACCATGATG	CGGATATGAGGCAGCAGTTTC	AGGGCACCTGGAAAACAACCCAGC
COL2A1	GGCAATAGCAGGTTCACGTACA	GATAACAGTCTTGCCCCACTTACC	CCTGAAGGATGGCTGCACGAAACATAC
RUNX2	AGCAAGGTTCAACGATCTGAGAT	TTTGTGAAGACGGTTATGGTCAA	TGAAACTCTTGCCTCGTCCACTCCG
COL10A1	ACGCTGAACGATACCAAATG	TGCTATACCTTTACTCTTTATGGTGTA	ACTACCCAACACCAAGACACAGTTCTTCATTCC
MMP13	CGGCCACTCCTTAGGTCTTG	TTTTGCCGGTGTAGGTGTAGATAG	CTCCAAGGACCCTGGAGCACTCATGT
COL1A1	CCCTGGAAAGAATGGAGATGAT	ACTGAAACCTCTGTGTCCCTTCA	CGGGCAATCCTCGAGCACCCT

Supplemental Table S2. List of assays on demand used for qPCR.

Gene	Assay ID
SOX9	Hs00165814_m1
ACAN	Hs01050178_m1
SP7	Hs00541729_m1
PPARG	HS00234592_m1
